# Multiple Systemic Infarctions as a Complication of Bronchial Artery Embolization With Polyvinyl Alcohol: A Case Report

**DOI:** 10.4021/jocmr665w

**Published:** 2011-09-26

**Authors:** Abdullah Ozkok, Ayse Serra Ucar, Timur Selcuk Akpinar, Gulfer Okumus, Esen Kiyan, Orhan Arseven

**Affiliations:** a Istanbul Faculty of Medicine, Department of Internal Medicine, Istanbul University, Istanbul, Turkey; b Istanbul Faculty of Medicine, Department of Chest Medicine, Istanbul University, Istanbul, Turkey

## Abstract

Bronchial artery embolization (BAE) is a widely used modality of treatment for massive hemoptysis which has well known complications such as chest pain, groin hematoma and transverse myelitis but systemic embolization of the material used in the procedure is very rare and was reported only four times in the literature which were all caused by the use of microspheres. Herein we report a case of a 41-year-old caucasian male who had a history of tuberculosis 10 years ago. He had BAE with polyvinyl alcohol (PVA) for the treatment of recurrent hemoptysis. After the procedure, the patient complained of severe pain on the left upper quadrant and bilateral costovertebral regions and in abdominal CT, multiple infarcts in the subcapsular area of the spleen and kidneys were observed. This is the first reported case of inadvertent systemic embolization secondary to BAE with PVA. Although the risk of systemic embolization is greater with microspheres, still potentially serious and life threatening complications may occur with PVA.

**Keywords:**

Bronchial artery emobolization; Complication; Polyvinyl alcohol

## Introduction

Bronchial artery embolization (BAE) is a well-accepted and effective form of treatment for massive and recurrent hemoptysis but some potentially life threatening complications including organ infarcts due to inadverent systemic embolization of material used in the procedure may occur. We present a case of multiple infarcts in the spleen and kidneys secondary to bronchial artery embolization with polyvinyl alcohol (PVA).

## Case Presentation

**Figure 1:**
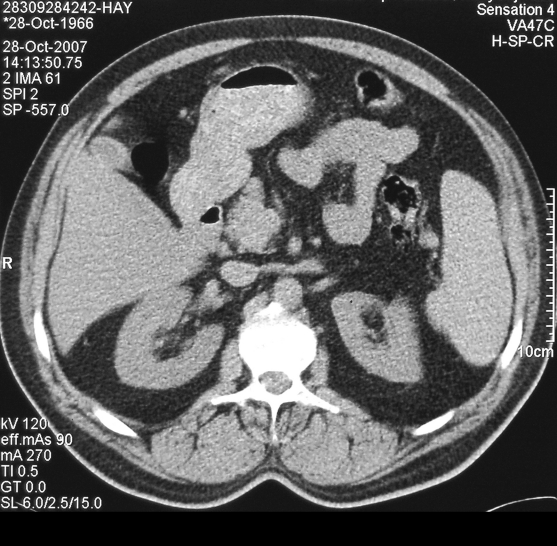
Abdominal CT is completely normal before the bronchial artery embolization.

A 41-year-old caucasian male who had a history of tuberculosis 10 years ago was referred to our clinic for BAE for the treatment of recurrent hemoptysis of 30-50 cc per day for the last 3 months. Thorax and abdominal CT were performed for the suspicion of a malignancy. Abdominal CT was totally normal (Fig. 1) and thorax CT demonstrated only fibrotic sequels and bronchiectatic lesions in the apical segment of the right upper lobe and pleuroparenchymal sequel band formations in the superior segment of the right lower lobe and no sign of a malignancy. The source of the hemoptysis was considered to be these sequel lesions secondary to past pulmonary tuberculosis.

**Figure 2:**
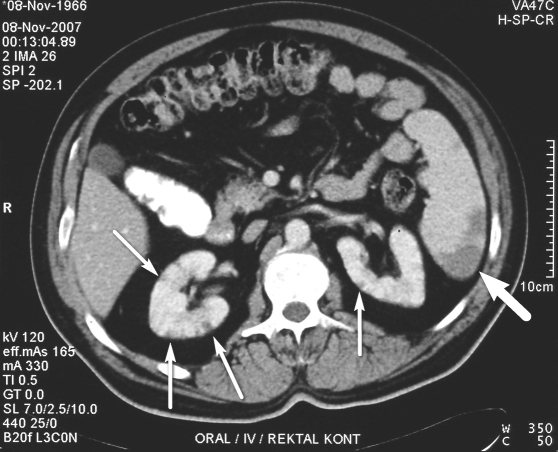
Abdominal CT shows multiple infarcts in kidneys (thin arrows) and spleen (thick arrow) just after the bronchial artery embolization.

Hypertrophic common bronchial artery trunk, right bronchial artery, and fourth intercostal artery were selectively catheterized and embolized with PVA in one session without intraoperative complication. On the same day after the procedure, the patient complained of severe pain on the left upper quadrant and bilateral costovertebral regions. Physical examination revealed fever, abdominal distension, left upper quadrant and bilateral costovertebral region tenderness, defence and rebound. Complete blood count showed neutrophilic leukocytosis (leukocyte, 14.200/ μL; neutrophil, 10.500/μL). C-reactive protein (CRP) was 122 mg/dL; lactate dehydrogenase (LDH) was 1193 U/L. Abdominal CT revealed multiple infarcts in the subcapsular area of the spleen and kidneys (Fig. 2). The electrocardiography (ECG) was normal and echocardiography showed neither an intracardiac thrombus nor a possible other source of systemic emboli. Therefore bronchial artery embolization was considered to be the cause of the multiple systemic infarcts. With conservative treatment, abdominal pain regressed and neutrophilic leukocytosis disappeared. High CRP and LDH levels were normalized. At follow-up, there was no deterioration of kidney functions. Control abdominal CT which was performed after 15 days of the embolization, showed regression of the infarcts.

## Discussion

BAE has various reported complications which may be severe and life threatening such as spinal cord infarction, transverse myelitis [1], myocardial infarction [2], serebrovascular accident [2-4], splenic and renal infarcts [3], esophagobronchial fistula [5], diaphragmatic paralysis [6], infarction of the bronchus [7], subintimal dissection of aorta [1,8], arterial perforation [8], mediastinal hematoma [1], or mild and self-limited such as chest pain [1,8], shoulder pain [8], transient dysphagia [8,9], transient cortical blindness [10], transient left orbital and forehead pain [9] and groin hematoma [8].

Systemic embolization during BAE is a very rare complication and to our knowledge there are only four reported cases including infarcts in the spleen and kidneys, myocardial infarction and serebrovascular accident [2-4]. Embolization materials used in these reports were microspheres. Our case is the first report of systemic embolization due to BAE with PVA.

We postulate that PVA had traversed from the bronchial circulation into the pulmonary circulation and then into small pulmonary arteriovenous malformations, and finally into the systemic circulation causing multiple infarcts in the spleen and both kidneys.

Actually, the risk of systemic embolization is higher with microspheres when compared with PVA because PVA has variability in size and a tendency to aggregate. These properties limit the ability to embolize peripheral vessels and cross arteriovenous anastomoses; however, microspheres do not have these limitations [3].

### Conclusion

We have presented the first case of inadvertent systemic embolization secondary to BAE with PVA. Although the risk of systemic embolization is greater with microspheres, still potentially serious and life threatening complications may occur with PVA.
